# Notes and Notices

**Published:** 1866-11

**Authors:** 


### NOTES AND NOTICES.

ONE of the most concise, comprehensive, truthful and practical articles we have ever read on the subject of CHOLERA, is found in the Boston Watchman and Reflector for September 27, 1866; the name of the author, or its source, ought to have been announced. The main positions are directly contrary to present received or fashionable opinions, and are almost in verbal accordance with the points insisted upon in the pages of this journal for the last twelve years, among these are,

FIRST. Quarantine is worse than useless, for it not only does not prevent the disease from visiting a point but diverts energies in a wrong direction, which if properly expended would have removed the causes which generate the malady.

SECOND. Cholera is not "catching," is not in any way communicated by one person to another, but arises in each individual from causes within himself, or belong to the locality in which he is.

THIRD. Intercommunication is not the law of the spread of Cholera, in that in multitudes of cases, it has overleaped localities in the direct line of travel, by a hundred miles or more, and at the end of weeks or months, has attacked the place which it had passed over.

FOURTH. That there are two causes of Cholera, a remote and an immediate, and both must be present at the same time, or the epidemic cannot exist.

FIFTH. That the remote cause is an influence about which we are as yet ignorant; that the immediate cause, that which excites the attack in every case is either personal or local.

SIXTH. The personal causes of an attack of Asiatic Cholera are over-eating, over-heating, over-working, apprehension, and such like.

SEVENTH. Local cause is uncleanliness, such as is connected with decaying substances, animal or vegetable, or both.

EIGHTH. That while we have no control over the remote causes, we do have an almost perfect control over the immediate; in that the personal causes are easily avoided, and the local can be removed or counteracted.

NINTH. That the whole subject is embraced in less than a dozen words; personal temperance, domiciliary cleanliness, bodily quiet and warmth in a bed.

TENTH. The immediate causes of Cholera are brought into operation by warmth and moisture acting on vegetable and animal dead substances, as evidenced by a fact of universal observation, that whenever a number of persons are attacked, it is always in a locality which is low, flat, damp and dirty.

ELEVENTH. The symptoms of an attack of Cholera in cholera times are forcible, large, watery, dirty, whitish colored passages from the bowels.

TWELFTH. The treatment—perfect quiet and warmth in bed, eat only ice if thirsty, and send for a physician.

Messrs. Broughton and Wyman, No. 13 Bible House, New York, the efficient agents of the "American Tract Society," at 28 Cornhill, Boston, Mass., have sent for notice "The Little Gold Keys," by Mrs, J. P. Ballard, 151 pp., encouraging the young to use means for the more instructive reading of the Bible, the oldest and the most precious of all books, being "the way, the Truth and the Life," to all who love to read, and study, and pray over it. Reader! are you one of these favored ones? then ought you to be "ever thankful." "The story of Zadoc Hull," 187 pp. It is a deeply engrossing story in connection with the war, and those who have lost children in the great strife, cannot fail to read and profit by it, as it helps to reconcile the ways of Providence to man, and does it so sweetly, too. "Recollections of Mary Lyons," with selections from her instructions to her pupils in Mt. Holyoke Female Seminary, by Fidelia Fisk, 333 pp., 12mo. "The Thirty-eight Selected Sayings to Teachers and some forty others for Pupils," are so full of wisdom, so obviously true, so widely suggestive, that we could wish the entire volume could be placed in the hands of every teacher and of every young girl in our land; it is a book full of practical Bible piety, and no Christian heart could fail to feed upon its contents; it merits a very wide circulation.

"Frank's Search for Sea Shells," by the author of "Rambles after Land Shells," 352 pp.; it is emphatically a book for all ages from six to sixty, as it leads the mind from the contemplation of nature to nature's God, and makes upon it an indelible impression of the wisdom and benevolence of Him who made all worlds, and that his kind eye and individual notice rest upon all that has been created, to sustain, to care for and preserve, from the inert atom to the winged Arch-Angel, and as a man is what the books he reads makes him, they are wise for both worlds whose selections are from publications of this class which instruct the mind, mature the affections, and elevate and sanctify the whole character.

"The Two Ways," by Catherine Bell, 64 pp., an instructive narrative for children, showing the immeasurable distance both for this world and that which is to come, between the destruction of him who walks in the hard way of the transgressor and of him who walks in the way of that wisdom which insures pleasantness and peace, a peace which the world can never give nor ever take away.

"Pictures and Lessons for Little Readers," 96 pp., contains a picture for every page, illustrating some useful, practical truths; a good present for good children.

The August No. of the *Social Science Review*, enlarged edition, monthly, $4 a year in advance — published at 84 Nassau St., N. Y., postage free, contains an admirable engraving of Herbert Spencer, and a sketch of his character. Also, Should taxation be compulsory? — The congregate system of Judicial Reformation. — Crime and Punishment. — What is Free Trade? Ladies' Financial Economy. — All written with ability, and are papers of sterling worth, meriting the patronage of educated men of all classes and parties.

One great and everlasting truth has been ineffaceably impressed on the public mind by the Press and by the hard logic of facts, in a manner never so thoroughly done before in all past history. It is this: That an atmosphere arising from filth of person, house and neighborhood is the prolific cause of cholera, in cholera times; and that the removal of it is the certain means of the immediate arrest of the spread of the disease. It is a practical lesson of incalculatable value.

SOUTHERN. — We long for the time when the streets of New York shall show the familiar faces of Southern men and women; the men the embodiment of all that was cordial, generous, and high minded; the women, beautiful, lovely and queenly, in whose tones of voice there was a sweetness, a warmth, a music, which at once won a high appreciation; there has been a dreadful war, but their natures are the same, and we believe that the individual feelings of persons to each other, North and South, have not changed. We do not think that there is a single man or woman, North, who, on meeting an old Southern acquaintance on Broadway, would not jump with delight to offer and receive the extended hand; and that both parties would overwhelm each other with kind inquiries of the olden time; the more cordial and affectionate for the long interim of darkness and desolation. This being so, we invite the patronage of the North to the sustenation of Southern publications, whose object is the promotion of learning and the spread of religious truth. Among others we name the *Medical Monthly* of Richmond, Virginia; the *Southern Review*, of New Orleans, and especially the Religious Weeklies; — *The Baptist*, published at Mount Lebanon, Louisiana, is just as good as it was before the War; and so is *The Southern Presbyterian*, of Columbia, S. C. *The Presbyterian Review* is conducted with ability and ought to be sustained. *The Christian Advocate* of Nashville, Tennessee, is one of the largest and best of the Methodist papers, and is full of valuable family reading.

best mode of reconstruction is for the North to hold out a helping sympathising hand, by being ready in every way to aid in the promotion of education, art, and religious truth. We really believe that within five years, "THE SOUTH" will be the most flourishing and prosperous country on the face of the globe.—Being Southern born and bred, we are not ashamed of our relationship, nor of the impartial record of the war. They are a brave people and a christian people, as they always were, as to the great body of them, and will one day show themselves capable of 'greater things than these.' In the great "Conflict" which is past, we never, for the thousandth part of a second, doubted of victory for the North, neither had we an atom of sympathy for the idea the South was fighting for, but we have a sympathy and an attachment for the people and for their country, which time nor circumstance can ever eradicate.

"The cause of Cholera cannot ascend any great height perpendicularly, nor affect persons living in the second or third stories of any good residence."

The above statement is designated as a "singular" one; we have been insisting on the main idea for a dozen years in these pages, embodying it in the statement that there is exemption from cholera, in cholera times, and in cholera localities, in proportion to the elevation at which persons lived above the general surface; and gave as proof, the observations made by a London Sanitary Board ten years ago;—and also that the immediate cause of cholera, which is that which arises from vegetable decay in warm weather, cannot ascend perpendicular elevations, as rocks or wells; and that the Eastern nations acted on this idea hundreds of years ago during plagues and epidemics, by living in the upper stories of buildings; not even going down to obtain family supplies, but drawing them up from the streets in baskets, supplied by the country people who come to town in the middle of the day when the miasmatic influences are not present to any special hurtful extent, it being worst at sunrise and sunset.

So we are beginning to know almost as much as they did in the dark ages.

Young children who live in cities during the hot weather would be almost exempted from Summer diseases if they lived wholly in elevated, good buildings, and were regularly and properly fed.

